# Older Kidney Transplant Patients Are Over Immunosuppressed Using Standard Protocols With Differential Sex-Based Complications

**DOI:** 10.1155/joot/5547629

**Published:** 2025-10-14

**Authors:** Inji Alshaer, Rachel K. Y. Hung, Sumoyee Basu, Gabrielle Goldet, Gareth Jones, Mark Harber, Raymond Fernando, Ciara N. Magee, Reza Motallebzadeh, Ben Caplin, Alan D. Salama

**Affiliations:** ^1^UCL Centre for Kidney and Bladder Health, Royal Free Hospital, London, UK; ^2^Anthony Nolan Histocompatibility Laboratories, Anthony Nolan Research Institute, London, UK

**Keywords:** CMV infection, elderly, immunosuppression, rejection

## Abstract

**Background:**

Increasing numbers of older patients are undergoing kidney transplantation. While there is evidence for both sex- and age-related immunological variations increasing the risks of immunosuppression (IS), few centers enforce age- or sex-specific IS adjustments.

**Methods:**

We investigated outcomes of 148 kidney transplants performed in our center between April 2009 and March 2019 in recipients aged > 60 years and compared them to outcomes in 272 younger recipients (divided into age groups 18–34, 35–49, and 50–60 years), matched for degree of human leukocyte antigen (HLA) sensitization (calculated reaction frequency, cRF), number of donor–recipient HLA mismatches, and cytomegalovirus (CMV) serostatus, all treated with the same IS protocol. Outcomes were time to (i) first episode of biopsy-proven acute rejection (BPAR), (ii) first CMV viremia within the first 6 months, (iii) incidence of any new-onset malignancy, and (iv) development of donor-specific anti-HLA antibodies (DSAs).

**Results:**

Overall rates of BPAR were highest in the recipients under the age of 35, but with no evidence of a difference between older age groups. Conversely, the risk of CMV viremia and malignancy was significantly higher in older recipients; in the > 60-year-old group, CMV viremia HR: 2.66 (95% CI: 1.49–4.75), and malignancy HR: 7.3 (95% CI: 1.7–31.10) versus the youngest group with little evidence was confounded by comorbidity or donor factors on multivariate analysis. The risk of CMV infection was most marked in the oldest female group, while the risk of malignancy was greatest in older males. The development of DSA was equal across all age groups.

**Conclusion:**

Our data indicate that older recipient age is associated with increased risk of CMV viremia and malignancy after transplantation, suggesting an age-associated vulnerability to IS, with the risk occurring mostly in older women and older men, respectively. These data support the need to develop age- and sex-specific protocol adjustments.

## 1. Introduction

The prevalence of chronic kidney disease (CKD) increases with age. In the United Kingdom, an estimated 13.5% of people aged between 65 and 74 years and just over a third of those over the age of 75 have CKD stage 3–5 [1], with the median age of patients presenting with end-stage kidney disease (ESKD) at around 65 years. Similarly, in the United States, approximately 40% of the ESKD populations are aged above 65 [2]. As a consequence, there has been a substantial increase in the proportion of older patients on active kidney transplant waiting lists in recent years [3].

Kidney transplantation remains the optimum renal replacement therapy for patients with ESKD [4–6], albeit with a reduced benefit in terms of survival advantage [4, 7–10] and a slightly increased risk of one-year graft loss for patients above the age of 65 [11] compared to younger recipients. Although increased recipient age is associated with increased mortality after kidney transplantation [11], death with a functioning graft [8, 12–14] is the dominant reason for graft loss in the elderly. In comparison to those aged 18–29 years, recipients aged over 65 have a sevenfold risk of death with a functioning graft [15], with cardiovascular disease (CVD) being the most frequent cause of death. While many older patients have a history of cardiovascular morbidity, including hypertension, diabetes, and hyperlipidemia, immunosuppressive drugs, in particular corticosteroids and calcineurin inhibitors (CNIs), may aggravate CVD risk after kidney transplantation [16]. Infection and cancer are the next two common causes of death in older recipients and, in many cases, are likely to reflect synergetic effects of maintenance immunosuppression (IS) combined with age-related immunosenescence [17], the latter being reflected by a decreased number of naïve T cells and impaired T-cell and B-cell responses [18–20].

In transplantation, both the immune response of the recipient and the immunogenicity of the donor organ change significantly with age [21]. A multivariate analysis of UNOS data reported that the frequency of acute rejection decreased with recipient age [22], while early graft and patient outcomes are comparable to those of younger patients [23]. Nevertheless, early rejection does increase the overall risk of death in older adults, in most cases as a result of infection associated with additional IS therapy [24]. The dynamic between IS and morbidity is influenced by changes in the pharmacokinetics and pharmacodynamics of immunosuppressive agents in the aging population, which has been well documented, particularly in kidney transplantation [25]. Moreover, the impact of sex on susceptibility to IS effects in the older recipients has been poorly studied, despite recognized differences in pharmacokinetics of commonly used drugs and variations in susceptibility to infectious diseases between the sexes [26, 27].

Our objective was to investigate if a standard immunosuppressive protocol resulted in features of over-IS, as judged by increased incidence of cytomegalovirus (CMV) viremia or other infections, as well as malignancy, alongside lower rates of alloantibody development and acute rejection, in older compared to younger recipients. Our center is one of the few that adopts a strategy of not using routine prophylactic treatment for CMV but instead manages recipients using pre-emptive CMV viral assessment, which provides a distinct opportunity to more accurately assess the rates of CMV viremia. This approach allows for the evaluation of natural control over viral replication prior to any initiation of antiviral therapy and facilitates a rigorous assessment of the impact of IS on CMV viremia, a surrogate marker of immunocompetence. We believe our data provide the impetus and rationale for a clinical trial of age- and sex-based modification of IS regimens for kidney transplant recipients.

## 2. Materials and Methods

### 2.1. Study Design and Participants

This was a single-center retrospective cohort study of first-time kidney-only transplants performed from April 1, 2009 to April 1, 2019. The cohort did not include any dual organ transplants. The study cohort comprises all patients who underwent kidney transplantation at our center between 2009 and 2019. Within this period, 204 patients were transplanted from 2009 to 2014, and 213 patients from 2015 to 2019. These intervals reflect the natural distribution of cases in our dataset and were not selected based on specific criteria. Reporting these time frames provides context for the analysis and allows consideration of potential temporal trends. Older recipients were defined as adults aged > 60 years at transplantation, based on an age of 60 being a cutoff for differing immunological outcomes and survival benefits in other studies [28–30]. We compared clinically defined outcomes (see below) in the older cohort to contemporaneous transplant controls matched for the number of human leukocyte antigen (HLA) donor–recipient mismatches (at HLA-A, B, and DR loci, 0–6), HLA calculated reaction frequency (cRF) (0%, 0%–85%, or > 85%), and CMV serostatus (recipient− or recipient+), across a range of younger age groups at the time of transplantation, subdivided into 18–34, 35–49, and 50–60 years of age. HLA antibody reaction frequency (or cRF) is calculated by comparison of unacceptable HLA specificities with HLA types of donors of identical ABO blood groups in a pool of 10,000 recent donors on the NHSBT database. Recipients with cRF ≥ 85% were classified as highly sensitized. We included only first transplants and recipients who had recorded blood results for at least 12 months post-transplantation and whose grafts survived for at least 3 months. Follow-up commenced at the time of transplantation and ended on January 31, 2022. Kidney transplant biopsies were performed only for clinical indications.

We manually matched older and younger transplant recipients from our database based on key factors that could influence doses of IS, such as immunological mismatch, immune reactivity, and CMV serostatus in donors and recipients. We did not use propensity matching or conduct a quasi-experimental study. Instead, our goal was to pragmatically observe how older patients fare when transplanted under a standard protocol.

Data for this observational cohort study were obtained via the Royal Free Hospital electronic records system and from the U.K. Transplant Registry of the Organ Donation and Transplant Directorate of National Health Service Blood and Transplant (NHSBT).

Clinical and laboratory data were collected retrospectively from electronic patient records. In accordance with U.K. National Health Service Research Ethics Committee guidelines, ethical approval was not required, as the study involved retrospective data and all treatment decisions were made prior to our evaluation.

### 2.2. IS Protocol

The immunosuppressive protocol described in this study reflects the local practice at our center and should not be considered representative of a standardized approach, as variations in protocols, including induction agents and maintenance therapies, are widely reported across different centers and international guidelines.

All patients received our standard IS using induction therapy with basiliximab (20 mg) given on the day of surgery and on the fourth postoperative day. Standard maintenance IS consisted of tacrolimus (started at 0.15 mg/kg/day, with target trough levels of 8–12 ng/mL in the first 3 months, 6–8 ng/mL in months 4–12, and 4–8 ng/mL after the 1st year), mycophenolate mofetil (MMF) (at 1 g twice daily for the first 3 months, reduced to 750 mg twice daily between months 3 and 12 and then tapered at 12 months to 500 mg twice daily), and early steroid withdrawal (methylprednisolone 40 mg once daily for the first 3 days, followed by prednisolone 20 mg for the next 7 days, reduced to 5 mg for one more week, then stopped). Importantly, prophylactic therapy for CMV prevention is not instituted at our center regardless of donor or recipient CMV status. Active monitoring of CMV viremia by twice-weekly plasma CMV DNA polymerase chain reaction (PCR) is employed for the first 3 months, then according to clinical indication. Antiviral therapy (valganciclovir) is initiated if detection of virus DNA is above a threshold of 200 and 3000 IU/mL in CMV-naïve and CMV-seropositive recipients, respectively. Recipients who were CMV IgG positive and who were treated with antithymocyte globulin therapy for steroid-resistant biopsy-proven acute rejection (BPAR) received universal prophylaxis with valganciclovir for 180 days followed by CMV PCR monitoring every 2 weeks for a minimum of 3 months. All patients received co-trimoxazole prophylaxis for 3 months, and valaciclovir prophylaxis is administered for 1 month to recipients who are herpes simplex virus (HSV) IgG negative. BKV DNA monitoring was carried out on blood three times a month in the first 12 months and then every 6 months for the subsequent year.

### 2.3. Outcome Variables

Outcome measures included graft loss, graft loss censored for death, occurrence of the first CMV viremia within the first 6 months, patient death, occurrence of BPAR, new-onset malignancy, and development of donor-specific anti-HLA antibodies (DSAs) by 1 year after transplantation. Incidence of CMV viremia was based on CMV DNA PCR, using a threshold of 200 and 3000 IU/mL in CMV-naïve and CMV-seropositive recipients, respectively. Acute humoral and cellular rejections were defined according to Banff classification [29]. Screening for anti-HLA antibodies was performed by flow cytometry using the xMAP (Luminex) platform, utilizing LAB Screen Mixed Bead kits (One Lambda, West Hills, CA, USA). Positive samples were tested on HLA Class I and Class II single antigen kits to define antibody specificities. A baseline mean fluorescence intensity (MFI) cutoff of ≥ 2000 was used to report pretransplant unacceptable antigens to NHS organ donation and transplantation (ODT); any donor-specific antibodies (DSA) ≥ 500 MFI were considered to be significant post-transplantation. We also assessed graft and patient survival. Graft survival was defined as the time from transplantation to graft failure (earliest of return to dialysis, graft nephrectomy, or re-transplantation), with censoring for death with a functioning graft or at last follow-up evaluation. Patient survival was defined as the time from transplantation to patient death.

Finally, we also collected data on BK virus infection, defined as a plasma BK virus level above 100 copies/mL or evidence of BK nephropathy on biopsy; delayed graft function (DGF) is defined as the need for dialysis within 14 days after transplantation and renal function using estimated glomerular filtration rate (eGFR), which was determined using the Modification of Diet in Renal Disease equation [31].

### 2.4. Statistical Analysis

Continuous variables are presented as mean ± standard deviation (SD) or median and interquartile range (IQR), depending on the distribution of the data. Categorical variables are expressed as percentages, with group comparisons made using the Pearson's *χ*^2^ test. For comparisons across multiple groups, analysis of variance (ANOVA) was used for normally distributed variables, and the Kruskal–Wallis test was applied for nonparametric data.

Survival outcomes, including time to death, graft loss, first CMV viremia, first malignancy, and first episode of BPAR, were analyzed using Kaplan–Meier (KM) survival curves. To assess associations between age-group and outcomes, we employed Cox proportional hazards (PHs) regression. Adjusted models accounted for potential confounders, including recipient factors (sex, race, primary renal disease, body mass index, smoking history, and length of dialysis), donor factors (age, sex, race, and deceased or live status), and transplant factors (cold ischemia time). Hazard ratios (HRs) and 95% confidence intervals (CIs) were calculated, and associations were considered statistically significant when the 95% CI for the coefficient did not include unity.

The Cox regression model used was a cause-specific model, as competing risks were not a significant concern for the primary outcomes. The PHs assumption was tested using the Schoenfeld residuals test, which indicated no violation of the assumption.

No formal adjustment for multiple testing (e.g., Bonferroni correction) was applied due to the exploratory nature of the study and the relatively small number of comparisons. However, we recognize that such adjustments may be necessary in future studies to confirm these findings.

Statistical analyses were conducted using SPSS Version 26 (SPSS Inc., Chicago, IL, USA) and GraphPad Prism v10.

## 3. Results

### 3.1. Baseline Characteristics of Participants

One hundred and forty-eight recipients older than 60 years receiving their first kidney transplant were identified. The number of kidney transplants performed in older patients remained relatively stable over the study period in our center (Figure S1), averaging 24% of our total deceased donor transplant activity in 2009–2014 and 27% in 2015–2019. The patients from the older transplant group were each matched with up to two control first-time kidney transplant recipients under the age of 60 (total of 272 patients), selected from at least two of three age groups (18–34, 35–49, and 50–60 years) identified from across each contemporaneous transplant year (Table 1), and matched by the total number of HLA-A, -B, and -DR mismatches, cRF level, and recipient CMV serostatus.

There were statistically significant differences in donor age between the groups, with older recipients having received kidneys from older donors, which is as expected from the U.K. National Allocation Scheme [32]. Additionally, although there were roughly similar proportions of recipients who received kidneys donated after brain death (DBD) among the four age groups, a higher proportion of older recipients (50–60 years) received kidneys donated after circulatory death (DCD), while a higher proportion of younger recipients received kidneys from live donors (Table 2).

The median follow-up duration was 87, 100, 66, and 65 months for the 18–34, 35–49, 50–60, and over 60-year-old age groups, respectively (*p*=0.005) (Table 1).

A total of 290 patients (69%) needed dialysis before transplantation, and the median time on dialysis for the different age groups is shown in Table 1. Diabetes and hypertension were the main causes of ESKD in the oldest recipients, and congenital and IgA nephropathies were the most common causes among the younger age group. Transplantation from CMV seropositive donors into seronegative recipients (CMV D + R−) occurred in 4%, 10%, 9%, and 8% in the age groups 18–34, 35–49, 50–60, and > 60, respectively. The majority of patients received basiliximab induction therapy except for 6 patients (two in the 50–60 age group and four in the over 60 age groups) who received alternative induction agents as part of the 3C study [33] and one who received ATG for a positive flow cytometric crossmatch.

### 3.2. Patient and Graft Outcomes

A total of 52 deaths occurred during follow-up, representing 12% of the overall cohort. Of these, 40 deaths (27%) occurred in recipients aged over 60 years and 12 deaths (4%) in those under 60 years. Among the deceased, 13 (9%) in the > 60 group and 9 (3%) in the < 60 group died with a functioning graft. Univariate analysis showed significantly lower patient survival in the > 60 age group compared to all younger age groups (Table 3).

However, in the multivariable Cox regression analysis—adjusting for key recipient and donor variables—the HRs for death in the 35–49 and 50–60 age groups were not significantly different from the > 60-year reference group, as evidenced by overlapping 95% CIs (Table 3). This discrepancy between univariate and multivariable results likely reflects adjustment for important covariates that may account for differences in survival. Furthermore, the Wald test in the adjusted model did not reject the null hypothesis, potentially due to limited statistical power to detect smaller but clinically meaningful differences, especially given the smaller sample sizes in younger age strata.

DGF was most common in the older recipients, affecting 40% and 30% of patients in the 50–60 and > 60 age groups, respectively. During follow-up, recipients aged 50–60 years experienced nearly twice the rate of graft loss compared to the 18–34 and 35–49-year groups. Five-year death-censored graft survival rates were 90.1%, 90%, 85%, and 69% for the 18–34, 35–49, 50–60, and > 60-year groups, respectively.

Univariate analysis demonstrated a strong association between older age and inferior graft survival, with an HR of 4.01 (95% CI: 1.81–8.90) for the > 60 age group. However, after adjusting for recipient sex, ethnicity, diabetes mellitus, BMI, smoking history, dialysis prior to transplantation, and donor characteristics (age, sex, and ethnicity), this association was substantially attenuated (Table 3).

Regarding acute rejection, univariate analysis revealed a significantly lower risk of BPAR in all age groups compared to the youngest cohort. This trend was more pronounced in the adjusted multivariable model. For recipients over 60 years, the unadjusted HR for BPAR was 0.50 (95% CI: 0.24–1.04), which further decreased to 0.23 (95% CI: 0.07–0.67) after adjustment (Table 3). The incidence of de novo class I or II DSAs, or an increase in pre-existing antibodies, occurred at similar rates across age groups; however, these measurements were taken at variable time points, limiting direct comparisons (data not shown).

**Figure 1:**
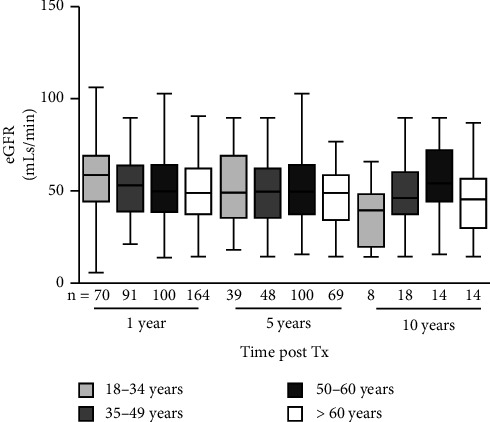
Box and Whisker plot (showing min and max range) of MDRD eGFR in each age group cohort at 1, 5, and 10 years after transplantation. Although the 1-year eGFR was statistically better in the younger cohort (*p*=0.002, one-way ANOVA), there was no difference at 5 or 10 years.

Median eGFR among patients with functioning grafts is shown in Figure 1. While younger recipients had significantly higher eGFR at 1 year, no differences were observed at 5 and 10 years post-transplant among surviving grafts across age groups.

To assess potential violations of the PHs assumption due to differing life expectancies across age strata, we performed a sensitivity analysis using a stratified Cox PHs model. Recipient age group (18–34, 35–49, 50–60, > 60 years) was used as the stratifying variable to allow for distinct baseline hazards, consistent with the Peto–Prentice methodology.

In this stratified model (see Supporting Tables 1 and 2), covariates such as BMI (HR 1.10, 95% CI: 1.009–1.208, *p*=0.032) and diabetes as a cause of ESKD (HR 0.294, 95% CI: 0.085–1.021, *p*=0.054) showed consistent associations with outcomes compared to the unstratified model. Dialysis prior to transplant trended toward significance (HR 2.47, 95% CI: 0.912–6.689, *p*=0.075), and donor age remained nonsignificant (HR 1.026, 95% CI: 0.991–1.061, *p*=0.143). These findings suggest that primary associations remained robust after accounting for non-PHs.

In the adjusted multivariable Cox model, recipient age was not significantly associated with graft survival. Nonetheless, KM survival curves demonstrated differences in graft survival distribution by age (Breslow *p*=0.021; Tarone–Ware *p*=0.024). The PH assumption was tested by including age as a categorical covariate, with no evidence of violation detected.

Notably, recipient ethnicity and diabetes mellitus as a cause of ESKD were independently associated with graft loss (*p*=0.031 and *p*=0.009, respectively), highlighting the importance of non-age-related factors such as metabolic comorbidities and ethnicity in long-term graft outcomes.

Finally, KM survival curves (Supporting Figures 2 and 3) further illustrated significantly different survival trajectories across age groups (log-rank *p* < 0.001), reinforcing the rationale for using a stratified modeling approach and supporting the overall robustness of our findings.

### 3.3. Infectious Complications

**Figure 2:**
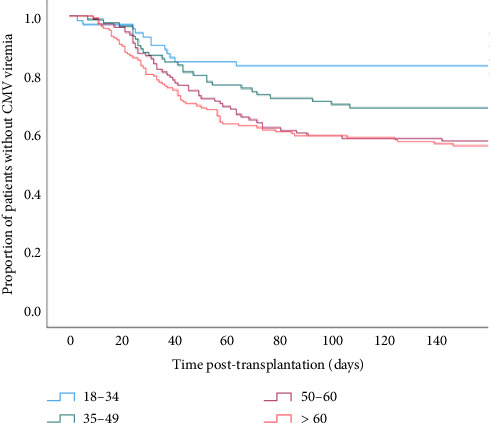
Kaplan–Meier curves for proportion of patients without CMV viremia within the first 6 months after transplantation in recipient age groups 18–34, 35–49, 50–59, and ≥ 60 (*p* < 0.003, log-rank test).

**Figure 3:**
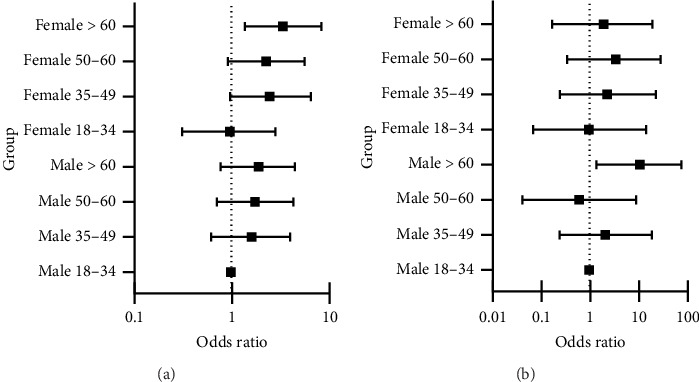
Forest plot of odds for developing (a) CMV according to age and sex and (b) risk of malignancy. Male recipients aged 18–39 were used as the reference group.

With regard to CMV infection, 44% of patients from the two older age groups (50–60 and > 60) developed at least one episode of CMV viremia in the first six months after kidney transplantation compared to 19% and 33% in the 18–34 and 35–49-year groups, respectively (Figure 2). There was no difference in the median time to developing CMV in the four age groups, and although the median peak CMV viral load was almost double in the > 60-year-old age compared to the youngest cohort, this did not reach statistical significance (Supporting Figure 4). Recipient age above 60 was associated with more than a twofold increased risk of CMV viremia (HR = 2.67; 95% CI = 1.50–4.75). The multivariate analysis demonstrated some attenuation of the HR, suggesting some of this risk may be confounded by donor and recipient factors other than their biological age (Table 3). However, there was a significant interaction between recipient age and sex (*p*=0.029) with female recipients more likely to become infected with CMV and older female patients at greatest risk, although this interaction effect was again attenuated in the adjusted model (Table 4) (Figure 3(a)). The rates of BK infection at any time post-kidney transplantation among the four age groups were not significantly different, but these were not always tested at standard times or consistently across the groups, making a time-dependent analysis more difficult (data not shown).

### 3.4. Malignancy

**Figure 4:**
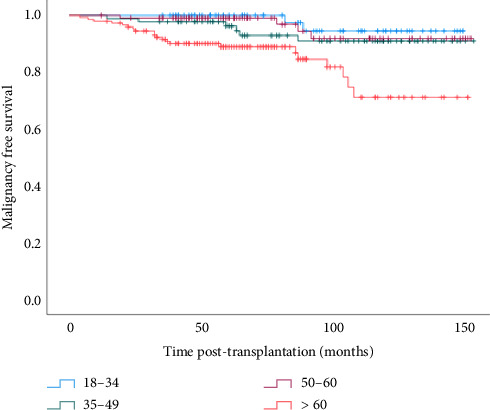
Kaplan–Meier curves for death-censored malignancy-free survival after transplantation in recipients in the age groups 18–34, 35–49, 50–59, and ≥ 60 (*p* < 0.001, log-rank test).

The most common malignancy reported was skin cancer (32%) for the whole cohort, with a new onset rate of 3% versus 15% in the 18–34 and > 60 age groups, respectively. The time to develop either skin, hematological, or solid organ malignancy in the four age groups is shown in Figure 4. Developing malignancy was significantly more common for recipients over 60 years HR 7.26 (1.69–31.10; Table 3) versus the youngest age group. This effect persisted in the multivariate analysis after adjustment for recipient and donor variables. There was a significant interaction between recipient age and sex, with the older male patients having the greatest risk of developing malignancy, which was somewhat attenuated in the adjusted model (Table 4) (*p* < 0.001) (Figure 3(b)). Examining cumulative incidence of cancer diagnosis (excluding non-melanoma skin cancers) showed a steady increase in the cancer rate over the 10 years post-transplantation across the four age groups, both for cases censored and not censored for graft loss, with the greatest incidence in the oldest age group (Tables 5 and 6).

## 4. Discussion

More than half of the deaths in older kidney transplant recipients are attributed to infection and malignancies [34]. Older subjects have decreased responses to vaccination, and there is increased progression of diseases associated with chronic inflammation and augmented rates of infection [35]. Sex differences in susceptibility to infection and response to vaccination have been reported but are variable, with both males and females being reported as more susceptible to certain infectious organisms [27]. The innate and adaptive immune systems, already dysregulated by age, are further disrupted by immunosuppressive medications, contributing to increased morbidity and mortality in kidney transplant recipients. In addition, there are numerous differences reported in the pharmacodynamics and pharmacokinetics of immunosuppressants between men and women [26]. Responses to bacterial infections are impaired in older transplant patients when compared to younger patients with identical regimens of induction and post-transplant immunosuppressive therapy; however, the relative impact on men and women has been little explored [36]. CMV infection in older kidney transplant recipients is associated with a higher mortality and is one of the risk factors for DGF and the requirement for hemodialysis post-transplantation in deceased donor transplant recipients [37–39]. In this study we demonstrate increased risks of CMV viremia and malignancies in our older patient population treated with similar immunosuppressive regimens. This was most significant for CMV in women and malignancies in men. This supports a more harmful impact of such standard immunosuppressive strategies in older rather than younger subjects and, importantly, demonstrates further susceptibility according to sex. However, the adjusted models suggest that there are other donor factors and recipient characteristics that also impact this susceptibility.

In our study, we found that younger kidney transplant recipients experienced more rejection episodes in comparison with the older kidney recipients. This is in keeping with previously reported data [23, 40]. In part this may be related to issues with drug compliance, which may be poorer in the younger age cohorts. In addition, there may be an impact from the lower frequency of pre-existing anti-HLA antibodies, both non-donor and donor-specific, in the older recipients. Moreover, de novo class II DSAs are found less frequently in older as compared to younger recipients [29]. The regression of B cell kinetics [41] and the innate immune system in the elderly is a possible explanation for this. While the presence of class II DSAs is associated with a higher rate of antibody-mediated rejection (AMR) in all transplant recipients [42], the rate of AMR was found to be lower in older recipients [41], reflecting the overall reduced antibody response in older patients.

Lower rates of cRF percentages were similarly found in the UNOS data set in older recipients [21], which is despite the fact that older patients would have been expected to have been exposed to more sensitizing events such as pregnancy, illnesses, and possibly blood transfusions during their lifetimes. Most likely, subdued sensitization stems from the aging immune system, although exactly when this occurs is difficult to ascertain due to the paucity of available data. However, we did not find any difference in the development of de novo DSA, but our study included only first kidney transplants, and we had matched the patients with younger cohorts with similar cRF, which might explain the lack of difference in the development of de novo DSA.

Older patients are also found to have a reduced response to interleukin-2 (IL-2) [43, 44]. It is therefore possible that the use of tacrolimus may be less effective in this patient group and that the exposure to deleterious side effects associated with CNIs is unnecessary. Using a mammalian target of rapamycin inhibitor like everolimus or sirolimus as a substitute for CNIs in transplant patients is an attractive substitute, and studies have shown that there is potential to improve outcomes post-transplantation in older patients [45, 46]. A recent phase III trial was in favor of an everolimus-facilitated tacrolimus minimization regimen of IS [47], and a study into the pharmacokinetics of this did not show any differences between younger and older kidney transplant recipients [48]. The use of basiliximab induction therapy may also be of less benefit in the older population due to an impeded response to IL-2.

What these data suggest is that as we transplant an increasing number of older patients, age- and sex-specific IS protocols could lead to improved patient and graft outcomes with reduced infections and malignancies without an increase in rejection in the aging immune system. This study is distinctive in that we sought to match older patients to younger patients based on their HLA mismatch, cRF levels, and CMV serostatus, aiming to reduce bias in likely alloimmune responsiveness. Our transplant unit is also unique in that all transplant patients receive the same IS protocol, and pre-emptive CMV therapy is not given regardless of donor or recipient CMV status. However, the study is small and needs to be replicated in larger cohorts.

Given that malignancy rates are expected to increase with age, we acknowledge that the higher malignancy rates observed in older patients are consistent with what is known in the literature. However, a more refined comparator, such as a cohort matched for age and comorbidities, could provide a more accurate assessment of malignancy risk. Future studies should aim to adjust for these factors to improve the interpretation of malignancy data in older patient populations.

The increased risk of infection and subsequent morbidity and mortality post-kidney transplant in the old kidney transplant recipients is likely to be due in part to the burden of IS. Therefore, there is an increasing need to establish guidelines on the usage of IS in older patients, especially when considering future infectious epidemics. In turn, such guidelines need to be informed by a robust evidence base, in the form of a clinical trial of modified IS in older recipients.

### 4.1. Limitations

This study has several limitations. First, it is a retrospective analysis, which is subject to the inherent issues of data acquisition and potential loss to follow-up. To mitigate this, we included only those patients with complete follow-up data.

Second, our matching strategy was limited to a few key factors. While we adjusted for additional variables in our analysis, we acknowledge that some important factors may still be unaccounted for, which could affect the results. Our strategy was to adjust for variables that might affect the use and doses of IS used that would impact the outcomes we were studying. While our dataset might not perfectly represent other older transplant populations with different patient characteristics, we believe these adjustments have minimized the impact of such differences on our results.

It is important to note that the causes of ESKD vary across age groups: Younger patients often have congenital or urological causes, whereas older patients more commonly have hypertension or diabetes. Because of this, matching by ESKD cause would be challenging and was not the focus of our study, as it is less relevant to understanding how IS affects infection risk. Similarly, factors like BMI may differ across age groups. After accounting for all these variables, we concluded that age itself is a critical factor beyond these other characteristics.

Third, the relatively small sample size and the low number of patients reaching clinical endpoints, such as CMV infection and malignancies, limit the statistical power of our analysis. Although significant findings were observed in the sub-analysis for CMV infection and malignancy, these should be interpreted with caution. The nonsignificant results from the overall multivariable model (Wald *p* value) suggest that the sub-analysis may reflect a spurious finding or be influenced by confounding factors. Therefore, these results require further validation in larger studies to assess their true significance.

Additionally, the small sample size limits our ability to detect smaller effects. Despite this, the study provides valuable preliminary insights into CMV infection risk. Larger studies with more participants are needed to validate these findings and explore smaller effects.

Another limitation is the absence of mean tacrolimus levels in our cohort. Our institution does not use age-based tacrolimus dosing protocols. Consequently, age-related adjustments in tacrolimus dosing were not considered in our analysis.

Although the protocol and tacrolimus level target differed across age groups, the actual immunosuppressive burden was not measured. As a result, the variability in immunosuppressive exposure among different age groups could not be fully assessed, potentially affecting the interpretation of the relationship between IS and outcomes. Additionally, there are no data on symptomatic CMV disease, such as CMV syndrome or end-organ disease. The absence of this information limits the clinical relevance of the findings, as it prevents a more comprehensive understanding of how overimmunosuppression might impact patient health through clinically significant complications like symptomatic CMV disease.

Finally, we did not include KDPI in our multivariable analyses due to limited data availability in our cohort. However, we recognize its relevance in assessing donor quality and plan to incorporate it in future studies when more comprehensive datasets are available or when specifically investigating its impact on outcomes.

## Figures and Tables

**Table 1:** Demographic and clinical characteristics of transplant recipients.

	Recipient age: 18–34 yrs (*n* = 71)	Recipient age: 35–49 yrs (*n* = 90)	Recipient age 50–60 yrs (*n* = 111)	Recipient age ≥ 60 yrs (*n* = 148)	*p* value^∗^
Recipient age, years median (IQR)	28 (24–31)	42 (39–46)	55 (62–58)	68 (65–71)	< 0.001
Male recipient sex, *n* (%)	35 (50)	49 (49)	70 (63)	101 (68)	0.03
Recipient ethnicity, *n* (%)					0.03
White	29 (41)	35 (39)	48 (43)	84 (57)	
Asian	22 (30)	26 (29)	32 (29)	42 (28)	
Black	20 (29)	29 (32)	31 (28)	22 (15)	
Cause of ESKD, *n* (%)					< 0.001
DM	3 (4)	6 (7)	21 (19)	31 (21)	
HTN	4 (6)	11 (12)	12 (11)	22 (15)	
IgA nephropathy	10 (14)	17 (19)	7 (6)	9 (6)	
Urological	9 (13)	4 (4)	6 (5)	7 (6)	
Congenital	12 (17)	10 (11)	3 (3)	5 (3)	
Vacuities	8 (11)	10 (11)	10 (9)	5 (3)	
ADPKD	1 (1)	8 (9)	9 (8)	11 (7)	
Others or unknown	24 (34)	24 (27)	43 (39)	58 (39)	
BMI, kg/m^2^ mean (SD)	24 (4.7)	26 (4.7)	27 (3.8)	26 (4.1)	< 0.001
HLA-A, -B, and -DR mismatches, *n* (%)					0.20
0–2	22 (31)	22 (24)	19 (17)	34 (23)	
3–4	43 (61)	53 (59)	81 (73)	94 (63)	
5–6	6 (8)	15 (17)	11 (10)	20 (14)	
Donor–recipient CMV IgG serostatus, *n* (%)					< 0.001
Pos–pos	30 (42)	48 (53)	47 (42)	72 (48)	
Pos–neg	3 (4)	9 (10)	10 (9)	12 (8)	
Neg–pos	18 (25)	19 (21)	42 (38)	56 (38)	
Neg–neg	20 (28)	9 (10)	11 (10)	8 (5)	
Unknown to positive	0	5	1 (1)	0	
Recipient's current history of smoking, *n* (%)	10 (14%)	15 (17%)	14 (13%)	15 (10%)	0.312
Recipient's previous history of smoking, *n* (%)	24 (34%)	34 (38%)	42 (38%)	77 (52%)	0.055
Pre-emptive transplantation, *n* (%)	24 (34)	28 (31)	28 (25)	40 (37)	0.60
Median dialysis duration, days (IQR)	456 (191–913)	775 (463–1669)	878 (394–1744)	943 (465–1682)	0.29
Sensitization at transplantation, *n* (%)					0.08
0–0	49 (69)	56 (62)	70 (63)	111 (75)	
1–70	21 (29)	24 (27)	33 (30)	33 (22)	
70–85	1 (2)	4 (4)	3 (3)	3 (2)	
> 85	0 (0)	6 (7)	5 (5)	1 (1)	
CMV titer, median (IQR) in those with detected viremia	1492 (428–2938)	4582 (1329–7991)	1172 (471–5020)	3251 (618–6925)	0.06
DGF, *n* (%)	17 (24)	20 (22)	45 (40)	45 (30)	0.02
Median time to graft loss, in months (IQR), in those with graft loss	49 (17–86)	61 (31–81)	48 (22–75)	49 (40–84)	0.698
Median length of follow-up, months (IQR)	87 (55–119)	100 (56–123)	66 (48–113)	65 (47–95)	0.005

*Note:* Continuous variables are shown as either mean (SD) or median (IQR), and categorical variables as absolute value (percentage). The Kruskal–Wallis test was used for continuous variables, and the Pearson chi-squared test was used for categorical variables. CMV, cytomegalovirus; ESKD, end-stage renal disease; HTN, hypertension; DBD, donation after brain death; DCD, donation after circulatory death.

Abbreviations: ADPKD, autosomal dominant polycystic kidney disease; ATG, antithymocyte globulin; BMI, body mass index; DGF, delayed graft function; DM, diabetes mellitus; DSA, donor-specific antibody; HLA, human leukocyte antigen; LD, live donor.

**Table 2:** Demographic and clinical characteristics of transplant donors.

	Recipient age: 18–34 yrs (*n* = 71)	Recipient age: 35–49 yrs (*n* = 90)	Recipient age: 50–60 yrs (*n* = 111)	Recipient age ≥ 60 yrs (*n* = 148)	*p* value
Donor age, years median (IQR)	41 (30–51)	45 (37–53)	52 (45–60)	60 (50–68)	< 0.001
Male donor sex, *n* (%)	45 (63)	53 (59)	60 (54)	94 (63)	0.43
Donor ethnicity, *n* (%)					0.16
White	47 (66)	54 (60)	81 (72)	114 (77)	
Asian	8 (11)	11 (12)	12 (11)	11 (7)	
Black	9 (13)	8 (9)	5 (5)	8 (6)	
Unknown	7 (10)	17 (19)	13 (12)	15 (10)	
Donor status, *n* (%)					< 0.001
LD	35 (49)	32 (35)	21 (19)	41 (28)	
DBD	28 (40)	36 (40)	46 (41)	59 (39)	
DCD	8 (11)	22 (25)	44 (40)	48 (33)	
KDPI, % Mean (SD)	20 (29)	15 (27)	36 (34)	42 (42)	< 0.001
KDRI, median (IQR)	1.02 (0.84–1.16)	0.92 (0.74–1.26)	1.14 (0.95–1.31)	1.48 (1.24–1.77)	< 0.001
CIT, mins Median (IQR)	336 (135–744)	601 (185–887)	616 (431–843)	637 (225–864)	0.11

*Note:* Continuous variables are shown as mean (SD) and categorical variables as absolute value (percentage). The Kruskal–Wallis test was used for continuous variables, and the Pearson chi-squared test was used for categorical variables. CMV, cytomegalovirus; ESKD, end-stage renal disease; DBD, donation after brain death; DCD, donation after circulatory death.

Abbreviations: CIT, cold ischemic time; DSA, donor-specific antibody; HLA, human leukocyte antigen; KDPI, kidney donor profile index; KDRI, kidney donor risk index; LD, live donor.

**Table 3:** Cox regression model for different patient outcomes.

Recipient outcome per age group (years)	Univariate HR (95% CI)	Multivariable HR (95% CI)^∗^	Multivariable HR (95% CI)^∗∗^
Graft loss			
18–34	Reference	Reference	Reference
35–49	0.84 (0.30–2.32)	0.49 (0.13–1.76)	0.35 (0.09–1.37)
50–60	1.22 (0.47–3.15)	0.67 (0.20–2.23)	0.46 (0.13–1.62)
≥ 60	2.38 (1.03–5.48)	1.33 (0.48–3.66)	0.88 (0.30–2.59)
	Wald *p*=0.02	Wald *p*=0.24	Wald *p*=0.29
Graft loss censored to patient loss			
18–34	Reference	Reference	Reference
35–49	0.95 (0.35–2.55)	0.49 (0.14–1.71)	0.45 (0.12–1.64)
50–60	1.88 (0.78–4.55)	1.02 (0.35–2.97)	0.85 (0.28–2.56)
≥ 60	4.01 (1.81–8.90)	1.54 (0.58–1.05)	1.31 (0.48–3.59)
	Wald *p*=0.00	Wald *p*=0.17	Wald *p*=0.28
CMV infection within 6 months post-KT			
18–34	Reference	Reference	Reference
35–49	1.82 (0.966–3.438)	1.28 (0.63–2.60)	1.47 (0.70–3.09)
50–60	2.55 (1.394–4.574)	1.95 (0.99–3.86)	1.89 (0.94–3.82)
≥ 60	2.66 (1.497–4.745)	2.09 (1.06–4.12)	2.33 (1.16–4.68)
	Wald *P*0.005	Wald *P*0.09	Wald *P*0.09
Patient death			
18–34	0.00 (0.00–3.87E + 145)	0.00 (0.00–2.50E + 175)	0.00 (0.00–3.46E + 176)
35–49	0.05 (0.01–0.23)	0.00 (0.00–7.67E + 160)	0.00 (0.00–2.69E + 168)0.94
50–60	0.29 (0.14–0.59)	0.295 (0.20–1.30)	0.51 (0.20–1.28)
≥ 60	Reference	Reference	Reference
	Wald *p* ≤ 0.001	Wald *p*=0.58	Wald *p*=0.57
Rejection			
18–34	Reference	Reference	Reference
35–49	0.40 (0.17–0.92)	0.30 (0.10–0.86)	0.22 (0.06–0.74)
50–60	0.32 (0.14–0.75)	0.18 (0.05–0.59)	0.09 (0.02–0.37)
≥ 60	0.50 (0.24–1.04)	0.30 (0.11–0.81)	0.23 (0.07–0.67)
	Wald *p*=0.03	Wald *p*=0.01	Wald *p*=0.002
Malignancy			
18–34	Reference	Reference	Reference
35–49	2.29 (0.46–11.35)	3.305 (0.30–35.39)	3.99 (0.33–47.86)
50–60	1.53 (0.28–8.39)	0.00 (0.00–2.52E + 192)	0.00 (0.00–3.10E + 128)
≥ 60	7.26 (1.69–31.10)	11.68 (1.4–97.35)	10.47 (1.14–96.01)
	Wald *p*=0.001	Wald *P*0.05	Wald *P*0.15

^∗^Recipient sex, ethnicity, BMI, and dialysis prior to transplant. Donor age, sex, and ethnicity, presence of DM, and current and previous smoking.

^∗∗^Recipient sex, ethnicity, BMI, and dialysis prior to transplant. Donor age, sex, and ethnicity, presence of DM, current and previous smoking, CIT, cRF, total MM, graft type, and donor–recipient CMV status.

**Table 4:** Cox regressions showing interaction between age and sex on risk of CMV infection and malignancy.

Recipient outcome per age group (years)	Univariate HR (95% CI)	Multivariable HR (95% CI)^∗^	Multivariable HR (95% CI)^∗∗^
CMV infection	*p*=0.029	*p*=0.42	*p*=0.25
18–34 male	Reference	Reference	Reference
18–34 female	0.95 (0.32–2.83)	1.14 (0.38–3.46)	0.92 (0.30–2.86)
35–49 male	1.58 (0.61–4.08)	1.39 (0.49–3.90)	1.01 (0.34–2.96)
35–49 female	2.45 (0.93–6.44)	1.36 (0.49–3.79)	0.55 (0.18–1.69)
50–60 male	1.76 (0.73–4.26)	1.92 (0.73–5.03)	1.11 (0.40–3.09)
50–60 female	2.22 (0.89–5.53)	2.33 (0.87–6.23)	1.73 (0.63–4.75)
> 60 male	1.84 (0.78–4.336)	2.06 (0.81–5.21)	1.42 (0.54–3.73)
> 60 female	3.32 (1.36–8.1)	2.53 (0.95–6.72)	1.62 (0.60–4.40)
Malignancy	*p* < 0.001	*p*=0.156	*p*=0.287
18–34 male	Reference	Reference	Reference
18–34 female	1.00 (0.63–16.04)	0.0 (0.00–0.00)	0 (0.00–0.00)
35–49 male	2.16 (0.23–20.78)	2.24 (0.18–28.5)	3.71 (0.22–63.36)
35–49 female	2.44 (0.25–23.42)	1.15 (0.07–19.84)	1.64 (0.08–35.4)
50–60 male	0.59 (0.04–9.50)	0 (0.00–0.00)	0 (0.00–0.00)
50–60 female	3.33 (0.35–32.08)	0 (0.00–0.00)	0 (0.00–0.00)
> 60 male	10.62 (1.42–79.74)	9.84 (1.13–85.95)	11.64 (1.02–133.03)
> 60 female	1.89 (0.17–20.8)	2.26 (0.20–25.61)	1.12 (0.07–17.17)

^∗^Recipient ethnicity, BMI, and dialysis prior to transplant. Donor age, sex, and ethnicity presence of DM, and current and previous smoking,.

^∗∗^Recipient ethnicity, BMI, and dialysis prior to transplant. Donor age, sex, and ethnicity, presence of DM, current and previous smoking CIT, cRF, total MM, graft type, and donor–recipient CMV status.

**Table 5:** Cumulative incidences of nonskin malignancies and melanoma at 1, 3, 5, and 10 years after transplantation not censored for graft loss.

**Year post-transplant**	**18–34 age group**			
	**New cases**	**Population at risk**	**Incidence rate**	**Cumulative rate**

1 year	0	71	0	0
3 years	0	71	0	0
5 years	0	71	0	0
10 years	2	71	0.028	0.028

**Year post-transplant**	**35–49 age group**			
	**New cases**	**Population at risk**	**Incidence rate**	**Cumulative rate**

1 year	1	90	0.011	0.011
3 years	2	90	0.022	0.033
5 years	3	90	0.033	0.066
10 years	5	90	0.055	0.121

**Year post-transplant**	**50–60 age group**			
	**New cases**	**Population at risk**	**Incidence rate**	**Cumulative rate**

1 year	0	110	0	0
3 years	1	109	0.009	0.009
5 years	1	105	0.009	0.018
10 years	3	103	0.029	0.047

**Year post-transplant**	**> 60 age group**			
	**New cases**	**Population at risk**	**Incidence rate**	**Cumulative rate**

1 year	2	147	0.013	0.013
3 years	7	139	0.050	0.063
5 years	8	128	0.062	0.125
10 years	9	113	0.079	0.204

**Table 6:** Cumulative incidences of nonskin malignancies and melanoma at 1, 3, 5, and 10 years after transplantation censored for graft loss.

**Year post-transplant**	**18–34 age group**			
	**New cases**	**Population at risk**	**Incidence rate**	**Cumulative rate**

1 year	0	71	0	0
3 years	0	71	0	0
5 years	0	70	0	0
10 years	2	68	0.029	0.029

**Year post-transplant**	**35–49 age group**			
	**New cases**	**Population at risk**	**Incidence rate**	**Cumulative rate**

1 year	1	90	0.011	0.011
3 years	2	90	0.022	0.033
5 years	3	89	0.033	0.066
10 years	5	84	0.059	0.125

**Year post-transplant**	**50–60 age group**			
	**New cases**	**Population at risk**	**Incidence rate**	**Cumulative rate**

1 year	0	110	0	0
3 years	1	107	0.009	0.009
5 years	1	103	0.009	0.018
10 years	3	100	0.03	0.048

**Year post-transplant**	**> 60 age group**			
	**New cases**	**Population at risk**	**Incidence rate**	**Cumulative rate**

1 year	2	147	0.013	0.013
3 years	7	137	0.051	0.064
5 years	8	126	0.063	0.127
10 years	9	103	0.087	0.214

## Data Availability

The data underlying this study include sensitive patient information and are therefore not publicly available due to patient confidentiality. Data may be available from the corresponding author upon reasonable request and subject to appropriate ethical approvals.

## Nomenclature

AMR: Antibody-mediated rejection
ATG: Anti-thymocyte globulin
DBD: Donation after brain death
DCD: Donation after circulatory death
BKV: BK virus
BMI: Body mass index
BPAR: Biopsy-proven acute rejection
DGF: Delayed graft function
DSA: Donor-specific anti-HLA antibodies
CKD: Chronic kidney disease
CMV: Cytomegalovirus
CNIs: Calcineurin inhibitors
CRF: Calculated reaction frequency
CVD: Cardiovascular disease
eGFR: Estimated glomerular filtration rate
ESKD: End-stage kidney disease
DNA: Deoxyribonucleic acid
HLA: Human leukocyte antigen
HRs: Hazard ratios
HSV: Herpes simplex virus
IL2: Interleukin-2
IQR: Interquartile range
IS: Immunosuppression
KM: Kaplan–Meier
MFI: Mean fluorescence intensity
MMF: Mycophenolate mofetil
NHSBT: National Health Service Blood and Transplant
ODT: Organ donation and transplantation
PCR: Polymerase chain reaction
SD: Standard deviation
UNOS: United Network for Organ Sharing

## Supporting Information

Supporting Figure 1: Graph showing the proportions of kidney transplants in recipients over 60 years of age according to the year during the study period. Supporting Figure 2: Kaplan–Meier curves depicting patient survival after kidney transplantation across age groups (18–34, 35–49, 50–59, and > 60 years) demonstrate significant differences (log-rank test, *p* < 0.0001). Supporting Figure 3: Kaplan–Meier curves illustrating overall death-censored graft survival rates at study completion for kidney transplant recipients across age groups (18–34, 35–49, 50–59, and ≥ 60 years) revealed significant differences (log-rank test, *p* < 0.001). Supporting Figure 4: Peak CMV viremia in recipients testing positive post-transplant, according to age groups 18–34, 35–49, 50–59, and ≥ 60, *p* value = 0.063 (one-way ANOVA). Supporting Table 1: Stratified Cox regression model results for patient survival (stratified by recipient age group). Supporting Table 2: Stratified Cox regression model results for graft survival (stratified by recipient age group). Supporting Appendix 1: Transplant unit survey.
