# Supplementary material for: Older Kidney Transplant Patients Are Over Immunosuppressed Using Standard Protocols With Differential Sex-Based Complications
Source: J Transplant. 2025 Oct 14;2025:5547629. doi: 10.1155/joot/5547629 (PMC12539993; doi:10.1155/joot/5547629)
Supplement: Supporting Information — Additional supporting information can be found online in the Supporting Information section. [file 5547629.f1.pdf]

Supplementary Figure 1.

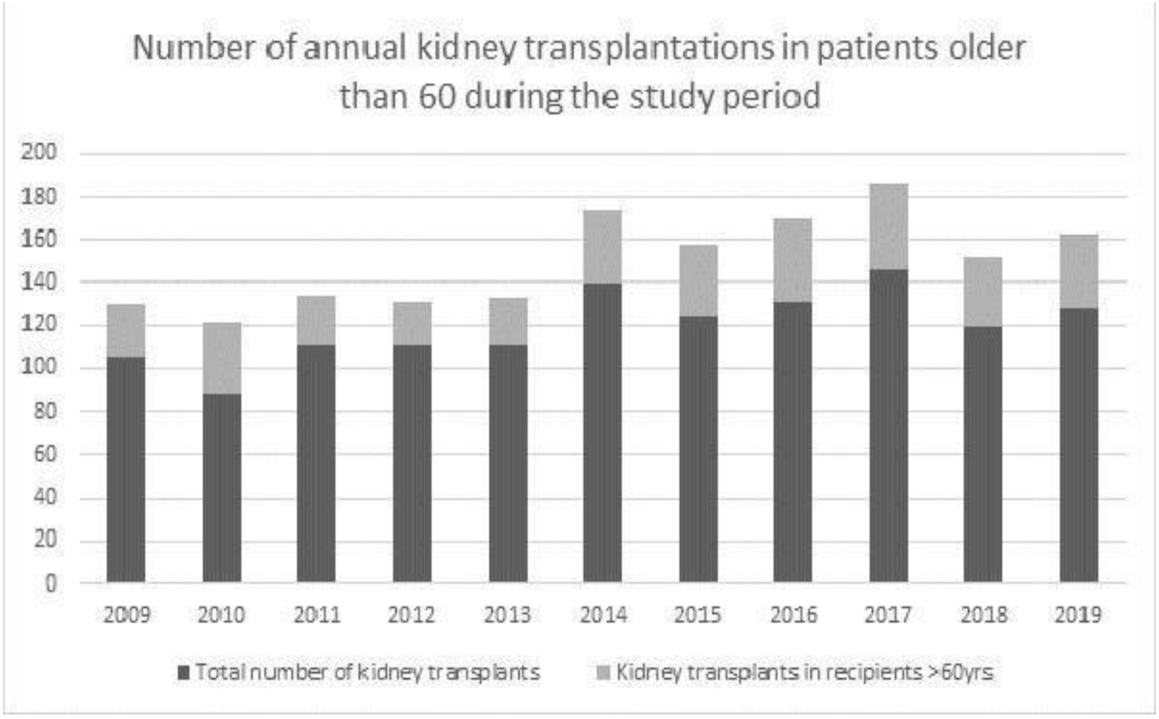

Supplementary Figure 2.

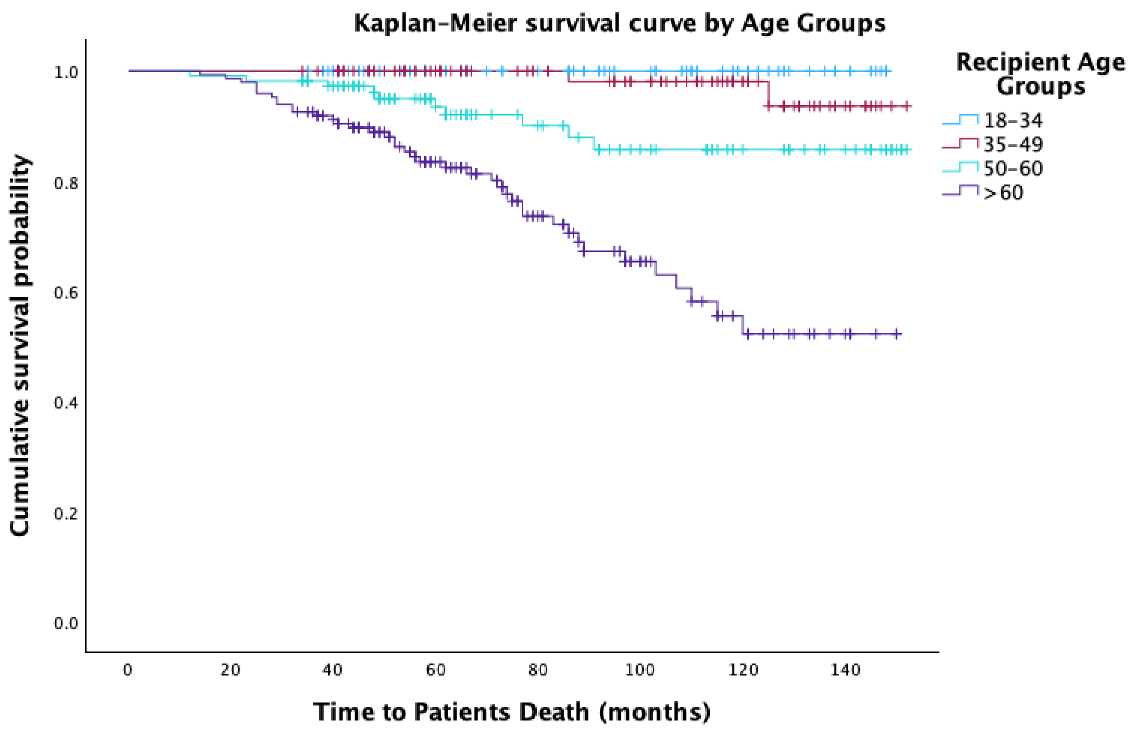

Supplementary Figure 3.

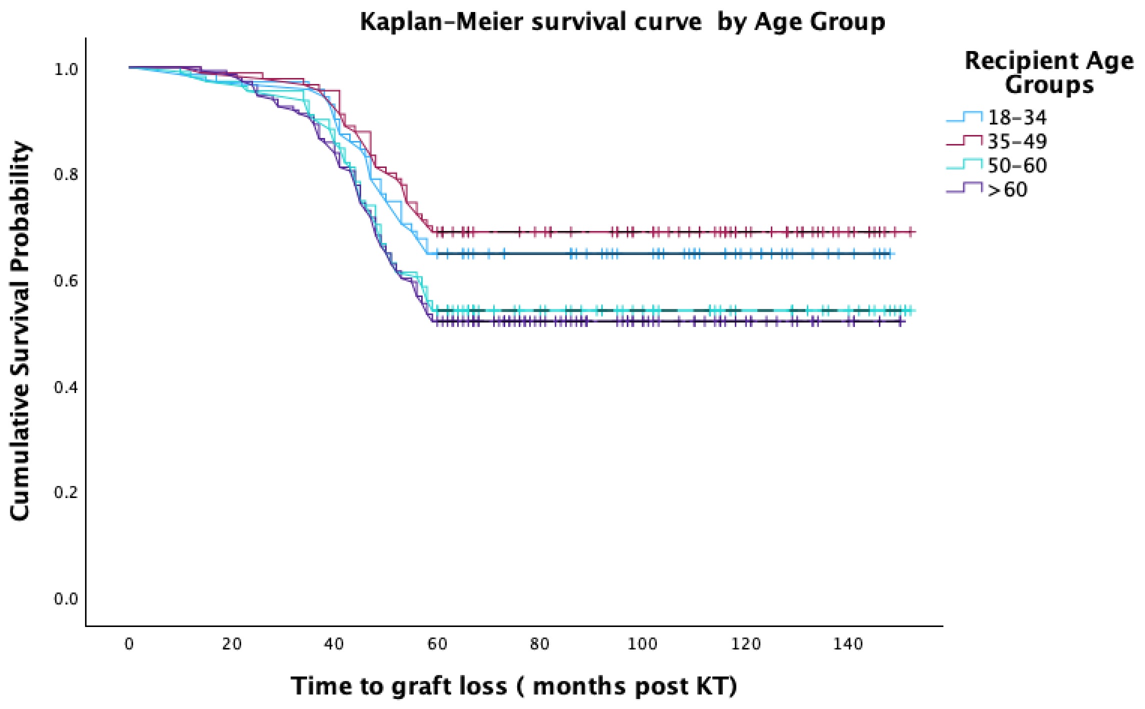

Supplementary Figure 4.

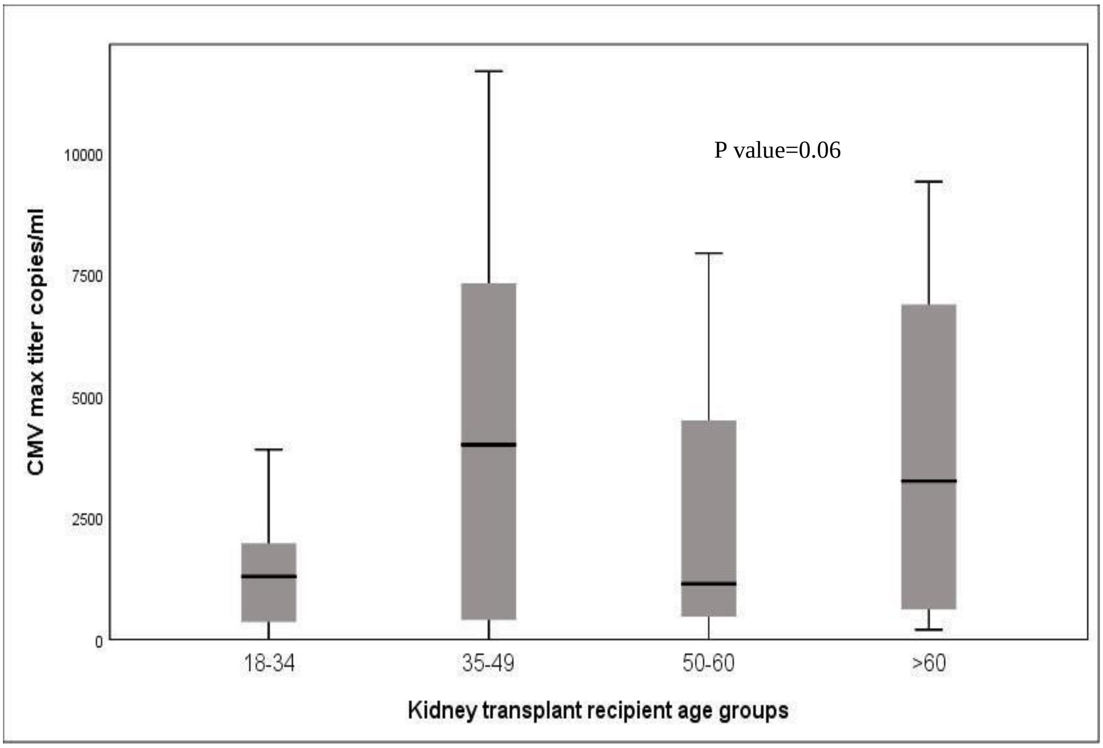

Table 1 : **Stratified Cox Regression Model Results for patients survival (Stratified by Recipient Age Group)**

Variable	B	SE	HR (Exp(B))	95% CI for HR	*p*-value
KT recipient gender (female vs male)	-0.133	0.481	0.875	0.341 – 2.249	0.782
KT recipient ethnicity (1)	-0.218	0.499	0.804	0.302 – 2.140	0.663
KT recipient ethnicity (2)	-0.145	0.595	0.865	0.270 – 2.774	0.807
Recipient BMI	0.099	0.046	1.104	1.009 – 1.208	0.032 ★
Dialysis pre-KT (yes vs no)	0.904	0.508	2.469	0.912 – 6.689	0.075
Donor gender (female vs male)	-0.089	0.377	0.914	0.437 – 1.913	0.820
Donor Ethnicity (1)	-0.166	0.774	0.847	0.186 – 3.865	0.830
Donor Ethnicity (2)	-1.401	1.124	0.246	0.027 – 2.232	0.213
Previous history of smoking	0.289	0.428	1.335	0.577 – 3.088	0.499
Current history of smoking	-0.368	0.649	0.692	0.194 – 2.469	0.571
DM as ESRD cause (yes vs no)	-1.223	0.635	0.294	0.085 – 1.021	0.054 †
Donor age (years)	0.025	0.017	1.026	0.991 – 1.061	0.143

Table 2 : **Stratified Cox Regression Model Results for graft survival (Stratified by Recipient Age Group)**

Variable	B	SE	Exp(B)	95% CI for Exp(B)	p-value
**KT recipient gender (female)**	-0.042	0.185	0.959	0.668–1.377	0.820
**KT recipient age group (ref: 18–34)**					0.401[1]
35–49	-0.318	0.296	0.727	0.407–1.300	0.283
50–60	0.105	0.268	1.111	0.657–1.880	0.695
>60	0.038	0.267	1.039	0.616–1.752	0.886
**KT recipient ethnicity**					0.067[1]
Ethnicity (1)	0.433	0.201	1.541	1.040–2.285	0.031 *
Ethnicity (2)	0.387	0.223	1.472	0.952–2.278	0.082
**Recipient BMI**	0.036	0.020	1.036	0.997–1.077	0.076
**Dialysis pre-KT**	0.291	0.195	1.338	0.914–1.959	0.136
**Donor gender (female)**	-0.114	0.227	0.893	0.643–1.238	0.495
**Donor ethnicity**					0.213[1]
Ethnicity (1)	-0.169	0.304	0.844	0.465–1.531	0.582
Ethnicity (2)	-0.699	0.411	0.497	0.222–1.112	0.089
**Smoking (previous history)**	0.293	0.195	1.341	0.915–1.965	0.129
**Smoking (current)**	-0.126	0.268	0.881	0.521–1.490	0.637
**DM as ESRD cause**	-0.769	0.295	0.463	0.260–0.826	0.009 **
**Donor Age**	-0.002	0.006	0.998	0.985–1.010	0.703

Supplementary appendix 1: Transplant unit survey

Immunosuppression in older renal transplant recipients (>65 years old)

Your transplant unit's use of immunosuppression

1. What is your induction regimen for renal transplantation at your unit? (select all that apply)

- ☐ Campath
- ☐ Simulect
- ☐ ATG
- ☐ MMF
- ☐ Tacrolimus
- ☐ Methylprednisolone
- ☐ Ciclosporin
- ☐ Azathioprine

Other (please specify) ________

2. If you have selected use of more than one monoclonal/polyclonal antibody, please explain when each is indicated?

3. What are your target calcineurin inhibitor levels post transplantation? (e.g. TAC, 0-3 months, target level 8-12 ng/ml; 3-12 months, target 6-8; over 12 months target 4-8)

4. What are your target MMF doses post transplantation?

5. Do you have any other target levels or doses for other immunosuppressive drugs post transplantation?

<u>Modifying immunosuppression in older recipients</u>

6. Do you change the immunosuppression (IS) regimen for the older transplant recipients at your transplant unit (at induction or post transplantation)?

Yes

No - skip to next page

7. Do you change the IS regimen at induction for older transplant recipients?

If yes, how?

If no, skip to next question

8. Do you change their IS regimen post transplantation?

If yes, how?

If no, skip to next question

Expert opinion

9. If your unit does not change the IS regimen for older recipients, do you think older transplant recipients should have a modified IS regimen?

Yes

No

10. Do you think a trial into reducing overall immunosuppression burden in older recipients is required?

Yes

No
